# Effectiveness of polaprezinc for low-dose aspirin-induced small-bowel mucosal injuries as evaluated by capsule endoscopy: a pilot randomized controlled study

**DOI:** 10.1186/1471-230X-13-108

**Published:** 2013-07-04

**Authors:** Ikue Watari, Shiro Oka, Shinji Tanaka, Taiki Aoyama, Hiroki Imagawa, Takayoshi Shishido, Shigeto Yoshida, Kazuaki Chayama

**Affiliations:** 1 Department of Gastroenterology and Metabolism, Graduate School of Biomedical & Health Sciences, Hiroshima University, Hiroshima, Japan; 2 Department of Endoscopy, Hiroshima University Hospital, Hiroshima, Japan

**Keywords:** Small-bowel mucosal injury, Low-dose aspirin, Polaprezinc, Capsule endoscopy

## Abstract

**Background:**

Treatment of low-dose aspirin (LDA)-induced small-bowel injury has not been established. Polaprezinc, a chelate of zinc and L-carnosine, may be efficacious for such injury. We conducted a pilot randomized controlled study to investigate whether polaprezinc is effective against LDA-induced small-bowel injuries.

**Methods:**

Consecutive patients under long-term (>3 months) LDA treatment and who agreed to participate in our study underwent initial capsule endoscopy (CE). Patients with LDA-induced small-bowel injury apparent upon initial CE (n = 20) were randomized into a polaprezinc (150 mg/day for 4 weeks) group and a control (no polaprezinc treatment) group. All underwent follow-up CE after 4 weeks. Changes in the number and characteristics of small-bowel mucosal injuries were compared within and between the two groups.

**Results:**

The median number of reddened lesions and erosions/ulcers upon follow-up CE in the polaprezinc group significantly decreased (P < 0.05). However, there was no significant difference in the median number of reddened lesions and erosions/ulcers upon follow-up CE in the control group.

**Conclusions:**

Co-administration of polaprezinc may be effective against small-bowel mucosal injury associated with long-term LDA therapy.

**Trial registration:**

UMIN Clinical Trials Registry UMIN000003687.

## Background

Aspirin, a nonsteroidal anti-inflammatory drug (NSAID), is one of the most-prescribed medications worldwide, and low-dose aspirin (LDA), usually defined as 75-325 mg daily, is commonly used for primary and secondary prevention of cardiovascular events and stroke [1-3]. However, the use of NSAIDs, including aspirin, is associated with risk of upper gastrointestinal mucosal damage, manifesting as peptic ulcer and/or bleeding [4-7]. NSAIDs, including aspirin, can also induce small-bowel injury and, now that small-bowel endoscopy is available for detection of small-bowel lesions, have become a matter of interest to gastroenterologists [8-10].

Neither protection against nor treatment of LDA-induced small-bowel mucosal injuries has been established. In Japan, polaprezinc is commonly used for the treatment of gastric ulcer. Polaprezinc is a chelate compound consisting of zinc and L-carnosine that is thought to function in protecting intercellular tight junctions [11,12], as an anti-oxidant [13], in preventing apoptosis [14-16], and in reducing inflammation [17]. Omatsu et al. [14] speculated that polaprezinc protects rat intestinal epithelial (RIE-1) cells from indomethacin-induced apoptosis via its reactive oxygen species (ROS)-quenching effect. Mahmood et al. [18] reported that zinc carnosine prevented the rise in gut permeability caused by indomethacin in healthy volunteers, strongly suggesting a small-bowel protective effect. Polaprezinc may have potential to protect against or treat NSAID-induced small-bowel injury. Therefore, we conducted a pilot randomized controlled study to assess the effectiveness of polaprezinc for treatment of LDA-induced small-bowel injuries.

## Methods

### Patients

Consecutive patients undergoing upper gastrointestinal endoscopy or colonoscopy at Hiroshima University Hospital and taking LDA between May 2010 and September 2011 were screened for inclusion in the study. These patients were visiting our hospital for endoscopic treatment of gastrointestinal tumor, follow-up endoscopic examination after endoscopic treatment, treatment of gastrointestinal bleeding outside the small intestine, or examination for obscure gastrointestinal bleeding (OGIB). Inclusion criteria were as follows: use of low-dose enteric-coated aspirin at 100 mg once daily for more than 3 months; no current use of antibiotics; age ≥ 21years; an initial CE examination. Exclusion criteria were as follows: use of NSAIDs other than LDA; inflammatory bowel disease; digestion-absorption disorder; polaprezinc treatment; use of other medicines for gastritis, e.g., misoprostol or rebamipide, within the prior 6 months; use of antibiotics or thyroxine sodium; stenosis of the gastrointestinal tract or severe adhesion; pregnancy or nursing; severe ulcerative lesion(s) observed upon initial CE, absence of small-bowel injury upon initial CE, and failure of the CE capsule to reach the cecum. Twenty patients in whom LDA-induced small-bowel injury was identified upon initial CE, as described below, comprised the final study group.

The study was conducted in accordance with the Declaration of Helsinki and was approved by the ethics committee of Hiroshima University Hospital. All patients screened for inclusion in the study were provided written informed consent for participation. The trial is registered with the UMIN Clinical Trials Registry as number UMIN000003687.

### Study protocol

The 20 patients in whom LDA-induced small-bowel injury was identified during the screening process were randomized into two groups: a polaprezinc group (n *=* 10) and a control group (n *=* 10). For allocation of patients to the study groups, we followed a block randomization scheme. Patients were given their assignments in sealed envelopes that had been shuffled previously. Patients in the polaprezinc group were given polaprezinc at 150 mg daily for 4 weeks, whereas patients in the control group were not given polaprezinc during the 4-week period. All patients continued LDA at 100 mg daily. Initial CE was defined as CE performed at the time patients were first examined in our department; follow-up CE was performed after 4 weeks in both groups.

### CE procedure and evaluation

The CE capsule (PillCam SB2; Given Imaging Ltd, Yoqneam, Israel) was swallowed with a solution of dimethicone after an overnight fast, without any other preparation. Images were analyzed with Rapid Reader 6.5 software on a RAPID 6.0 workstation (both from Given Imaging). CE images were reviewed independently by two of four experienced gastroenterologists (I.W., T.A., H.I., T.S.) who were not provided any clinical information. If the gastroenterologists’ findings differed, consensus was reached through discussion.

NSAID-induced small-bowel injury is characterized by multiple petechiae/red spots, denuded area, scars, mucosal erosions, and ulcers with a round, irregular, or punched-out appearance, or circumferential ulcers with stricture [19]. Such LDA-induced small-bowel injury was diagnosed at the time of initial CE.

**Figure 1:**
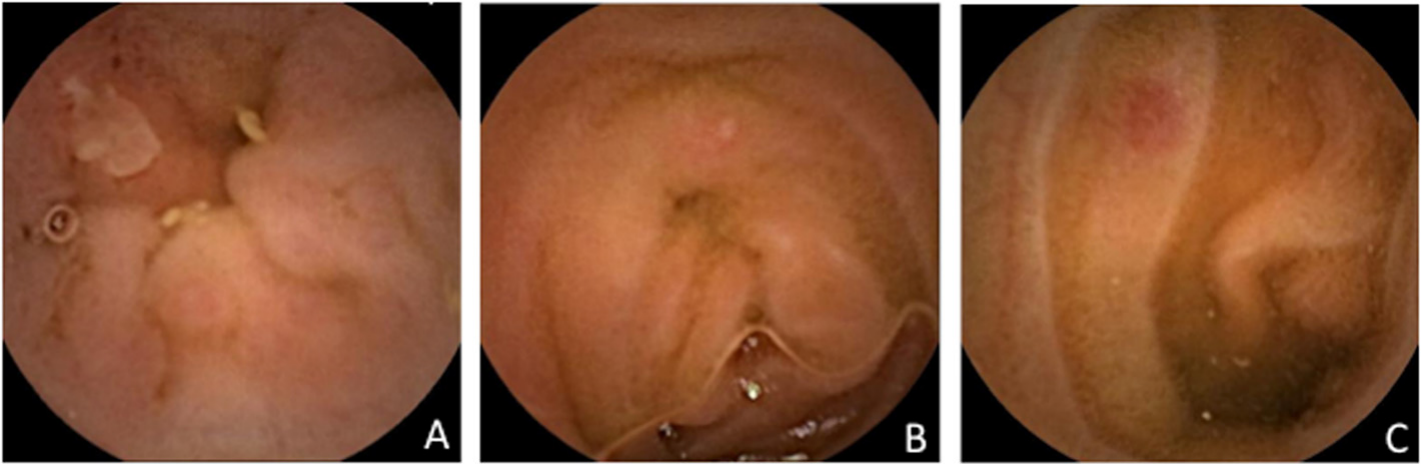
**Capsule endoscopy images of small-bowel mucosal injuries induced by low-dose enteric-coated aspirin therapy.** (**A**) Ulcer (depression with a white coating), (**B**) erosion (white spot surrounded by a red halo), (**C**) reddened lesion (reddish mucosal change).

Small-bowel mucosal injuries were classified as either erosion/ulcer or reddened lesion [20,21] as follows: erosion, a white spot surrounded by a red halo; ulcer, depression with a white coating; reddened lesion, reddish mucosal change such as reddened folds, denuded area, and/or petechiae (Figure 1); red spots were ignored in this study. LDA-induced small-bowel injuries were defined as follows; (1) ulcer, erosion or reddened lesion detected by capsule endoscopy, (2) use of LDA for more than 3 months, and (3) exclusion of Crohn’s disease, intestinal tuberculosis and any other small bowel disease.

We assessed the anatomic distribution of LDA-induced small-bowel mucosal injuries upon initial CE according to the following numerical formula based on capsule transit time through the small intestine [22]: Lesion location, i.e., first, second, or third portion of the small bowel corresponding to the percentage of total transit time, = (CE time when lesion is found – CE time of first duodenal image)/small bowel transit time.

To evaluate the effectiveness of polaprezinc, the number of small-bowel injuries and CE score [23] were calculated for each patient upon initial CE and upon follow-up CE. Changes in the number of small-bowel injuries and the difference in CE scores between initial CE and follow-up CE were compared between the polaprezinc group and the control group.

The CE score [23] was determined for small-bowel mucosal inflammatory changes. The score was based on three capsule endoscopy variables: villous appearance, ulceration, and stenosis. Severity of the mucosal inflammatory changes was assessed in tertiles by dividing small-bowel capsule transit time into three equal allotments. The total CE score was taken as the highest tertile score plus the stenosis score. The results were classified into three categories based on the final numerical score: normal or clinically insignificant change (total score <135), mild change (total score ≥135 but <789), and moderate to severe change (total score ≥790). This scoring system has been shown to be useful for evaluating aspirin-associated small-bowel mucosal disease activity and for objectively scoring the small-bowel inflammatory disease state [24].

### Statistical analysis

Quantitative data are presented as median (range), and categorical data are presented as the number per group. Between-group differences in sex ratio, anti-ulcer drug use, indications for LDA therapy, and tertile CE scores were analyzed by Fisher’s exact test. Within-group differences between initial CE and follow-up CE in the number of ulcers/erosions and reddened lesions and in CE scores were analyzed by Wilcoxon signed-rank test. *P* < 0.05 was considered statistically significant. All analyses were performed with JMP-J software.

## Results

Characteristics of the 20 patients randomized to the polaprezinc group or control group are shown per group in Table 1. There was no significant difference between the two groups with regard to sex ratio, age, hemoglobin concentration, indications for LDA, duration of LDA, or anti-ulcer drug use. Neither was there a significant difference between the two groups upon initial CE in the number of erosions/ulcers and reddened lesions, total CE score, or tertile CE scores.

**Table 1:** Characteristics of patients with small-bowel mucosal injuries per study group (polaprezinc treatment and no polaprezinc treatment, i.e., control)

**Characteristic**	**Polaprezinc (n = 10)**	**Control (n = 10)**	***P *value**
Sex ratio (male/female)	9/1	7/3	N.S.*
Age (years); median (range)	78.5 (64-82)	75.5 (62-86)	N.S.**
Hemoglobin concentration (g/dL); median (range)	13.6 (10.2-15.3)	13.7 (8-15)	N.S.**
Indication for low-dose aspirin therapy			
Valvular heart disease	3	2	
Stroke	3	7	N.S.*
Other	4	1	
Duration of low-dose aspirin (months); median (range)	64.5 (24-120)	48 (36-120)	N.S.**
Anti-ulcer drug			
H2 blocker	1	2	N.S.*
PPI	3	1	
None	6	7	
Initial CE findings			
Median number of erosions/ulcers (range)	2 (0-6)	2 (0-10)	N.S.**
Median number of reddened lesions (range)	3 (0-7)	2 (0-7)	N.S.**
CE score			
Median score (range)	180 (0-450)	225 (0-225)	N.S.**
CE score by category			
Normal or clinically insignificant change (<135)	3	4	
Mild change (≥135 and <790)	7	6	N.S.*
Moderate or severe change (≥790)	0	0	

Number of patients are shown unless otherwise indicated.

*by Fisher’s exact test **by Wilcoxon test.

Abbreviations: *CE*, Capsule endoscopy; *PPI*, Proton pump inhibitor; N.S., Not significant.

**Figure 2:**
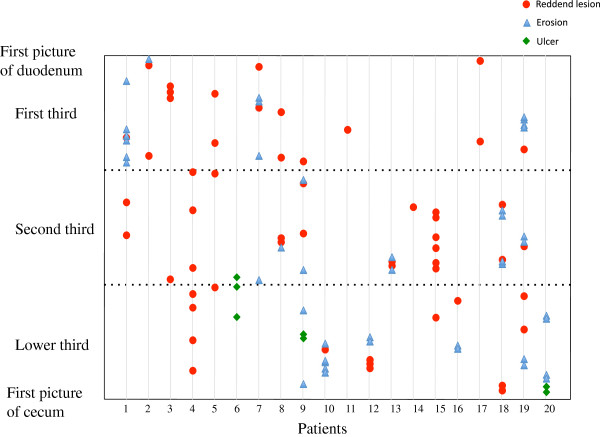
**Anatomic distribution of small-bowel mucosal injuries observed by initial CE.** Injuries are shown as one of three types for all subjects. Location (first, second, or third portion of the small intestine) of each injury was determined according to time the injury was observed in relation to total capsule transit time through the small intestine.

The types and anatomic locations of the small-bowel mucosal injuries observed upon initial CE are shown in Figure 2. Reddened lesions and erosions were evenly distributed throughout the small bowel, but ulcers tended to be located in the third section of the small bowel.

**Figure 3:**
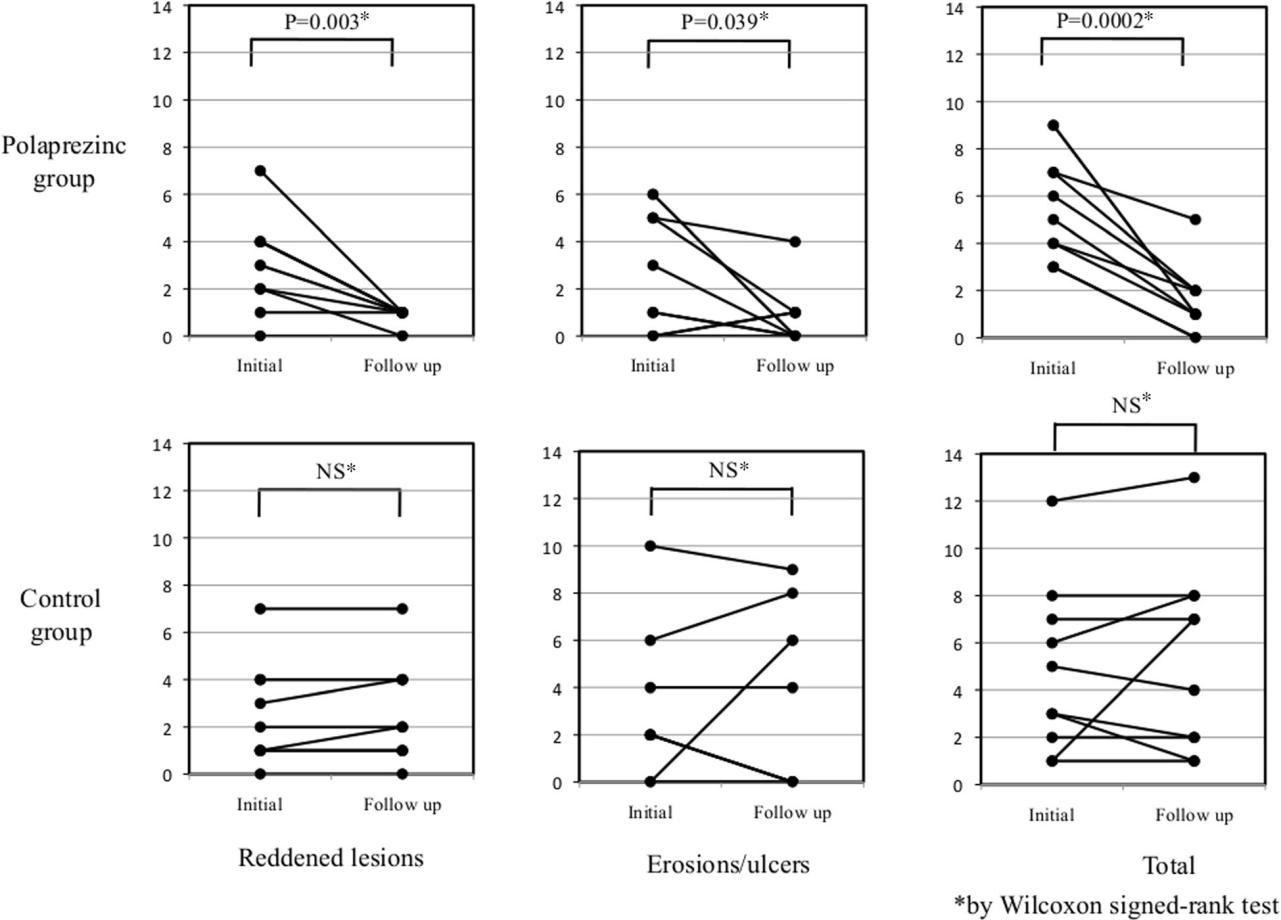
**Changes in the numbers of small-bowel mucosal injuries (from initial to follow-up capsule endoscopy).**

The median number of erosions/ulcers identified upon initial CE in the polaprezinc group was 2 (range 0-6), and the median number of reddened lesions was 3 (range 0-7). The median numbers in the control group were 2 (range 0-10) and 2 (range 0-7), respectively. As shown in Table 2, the median number of erosions/ulcers depicted upon follow-up CE in the polaprezinc group decreased significantly to 0 (range 0-4) (*P =* 0.039). In the control group, there was no significant difference in the median number of erosions/ulcers upon follow-up CE. The median number of reddened lesions in the polaprezinc group decreased significantly to 1 (range 0-1) upon follow-up CE (*P =* 0.003), but in the control group, there was no significant difference in the median number of reddened lesions observed upon follow-up CE. Change in the numbers of small-bowel mucosal injuries from initial CE to follow-up CE in both groups is diagrammed in Figure 3. In addition to the median numbers of reddened lesions, erosions/ulcers, the median number of total lesions (reddened lesions/erosions/ulcers) decreased significantly in the polaprezinc group. The changes in median CE score between initial CE and follow-up CE did not differ between the two groups.

**Table 2:** Number of small-bowel mucosal injuries and CE scores upon initial and follow-up CE in the polaprezinc treatment group and non-polaprezinc (control) group

	**Initial CE**	**Follow-up CE**	***P *value**
Polaprezinc group (n = 10)			
Median number of erosions/ulcers (range)	2 (0-6)	0 (0-4)	0.039*
Median number of reddened lesions (range)	3 (0-7)	1 (0-1)	0.003*
Median CE score (range)	180 (0-450)	0 (0-225)	N.S.*
CE score by category			
Normal or clinical insignificant change (<135)	3	6	
Mild change (≥135 and <790)	7	4	N.S.**
Moderate or severe change (≥790)	0	0	
Control group (n = 10)			
Median number of erosions/ulcers (range)	2 (0-10)	0 (0-9)	N.S.*
Median number of reddened lesions (range)	2 (0-7)	2 (0-7)	N.S.*
Median CE score (range)	225 (0-225)	0 (0-450)	N.S.*
CE score by category			
Normal or clinically insignificant change (<135)	4	6	
Mild change (≥135 and <790)	6	4	N.S.**
Moderate or severe change ((≥790)	0	0	

Abbreviations: *CE*, Capsule endoscopy; *N.S*., Not significant.

*by Wilcoxon signed-rank test **by Fisher’s exact test.

## Discussion

NSAIDs, including aspirin, cause small-bowel injury through cyclooxygenase (COX)-dependent and COX-independent pathways [25]. NSAIDs inhibit mucosal prostaglandin (PG) synthesis by inhibiting COX activity. NSAIDs decrease mucosal endogenous PG, resulting in reduction of intestinal mucus, microcirculatory disturbances accompanying abnormally increased intestinal motility, disruption of intercellular junctions, and increased mucosal permeability. Bjarnason et al. [26] proposed a “three hit” hypothesis independent of the COX-pathway. NSAIDs solubilize phospholipids on the mucosal surface, directly damaging epithelial mitochondria. The mitochondrial damage leads to calcium efflux and to induction of free radicals; disruption of intercellular junctions occurs, and mucosal permeability increases in the small intestine [27]. Mucosal injuries can be caused by the penetration of bile acid, proteolytic enzymes, intestinal bacteria, and/or toxins.

The reported incidence of LDA-induced small-bowel injury is 20-61.5% among healthy volunteers using short-term LDA [24,28-30], and the reported prevalence of LDA-induced small-bowel injury is 42.1-100% specifically among patients with OGIB using long-term LDA [19,24,31,32]. The actual overall clinical prevalence of adverse effects of long-term aspirin on the small bowel in asymptomatic patients remains undocumented.

Endo et al. reported that aspirin-associated small bowel ulcers tended to be located in the distal part of the small bowel [19]. In our patients, although there was no specific anatomic distribution of reddened lesions and erosion, ulcers were specifically found in the ileum upon initial CE, albeit there were only three such cases.

The recommended treatment for small-bowel injury in patients undergoing LDA therapy is withdrawal of the aspirin. However, in the majority of patients, LDA is used as an antiplatelet agent, and it cannot be discontinued due to the increased risk of cardiovascular or cerebrovascular morbidity and mortality. Prevention and healing regimens for LDA-induced small-bowel injuries are needed. To date, several investigations regarding prevention and healing regimens for aspirin-induced small-bowel injury have been reported. Watanabe et al. [32] reported that among patients suffering from cerebral and cardiovascular disorders, misoprostol was administered to those taking LDA for 3 months or more, and, after 8 weeks, CE revealed that red spots as well as mucosal breaks were completely eliminated in 57% of the patients. Unfortunately, there was a high incidence of side effects, e.g., diarrhea, hepatic dysfunction, and albuminuria, and misoprostol is generally contraindicated for women who could be pregnant.

Endo et al. [31] randomized patients taking LDA for 3 months or more into two groups: one given 3-month probiotic treatment (oral *Lactobacillus casei*) and the other not given the 3-month probiotic treatment. CE at 3 months showed that the number of reddened lesions and/or mucosal breaks had decreased in the group given *L. casei.*

The study reported herein is the first randomized controlled study of the effectiveness of polaorezinc on small bowel injury identified by CE in chronic LDA users. Although our study was small (20 patients), limited to a single center, and without a placebo control group, the median number of reddened lesions and erosions/ulcers in those treated with polaprezinc decreased significantly, suggesting that polaprezinc may be clinically effective in treating LDA-induced small-bowel injuries. No polaprezinc-based improvement in CE score was observed in our patients, but this might be due to the small number of ulcers among our study patients, to the fact that the lesions were fairly small, with most being less than one-quarter of the circumference of the small bowel, and to the absence of stenosis.

Generally, small-bowel endoscopy, including CE and DBE, is not performed except in OGIB cases or symptomatic cases. We are convinced that further studies are warranted to determine which patients undergoing LDA therapy should undergo endoscopic examination for small-bowel lesions, which drugs are effective for such lesions, and whether the same drugs can be used even to prevent such lesions.

## Conclusions

Co-administration of polaprezinc may be effective for LDA-induced small-bowel injuries.

## Competing interests

The authors declare that they have no competing interests.

## Authors’ contributions

IW analyzed the capsule endoscopies, collected the clinical data and wrote the manuscript, with contributions from SO, ST and KC. SO was responsible for the design of the study and collected the clinical data. SY performed the statistical analyses. TA, HI and TS analyzed the capsule endoscopies. All authors read and approved the final manuscript.

## Pre-publication history

The pre-publication history for this paper can be accessed here:

http://www.biomedcentral.com/1471-230X/13/108/prepub
